# Outcomes of a Management Strategy in Eyes with Corneal Irregularity and Cataract

**DOI:** 10.1155/2016/8497858

**Published:** 2016-08-03

**Authors:** Mathew Kurian Kummelil, Rohit Shetty, Luci Kaweri, Shama Shaligram, Mukesh Paryani

**Affiliations:** Narayana Nethralaya Eye Hospital, 121/C Chord Road, Rajajinagar 1st R Block, Bangalore 560010, India

## Abstract

*Purpose*. To evaluate the outcomes of a management strategy in patients with irregular corneas and cataract.* Methods*. Six eyes of four patients presented for cataract surgery with irregular corneas following corneal refractive surgery. Topoguided ablation regularised the cornea, followed by phacoemulsification and intraocular lens implantation. Zonal keratometric coefficient of variation (ZKCV) measured structural changes and visual quality metrics measured functional improvement.* Results*. The mean duration after corneal refractive surgery was 7.83 ± 2.40 years. The logmar uncorrected distance visual acuity (0.67 ± 0.25) and the corrected distance visual acuity (0.38 ± 0.20) improved to 0.34 ± 0.14 and 0.18 ± 0.10, respectively. The changes in the standard deviations of the zonal keratometry values and the ZKCV were statistically significant in the 2, 3, and 4 mm zones. The changes in the Strehl ratio (ANOVA *p* = 0.043) were also statistically significant.* Conclusions*. Corneal regularisation followed by phacoemulsification resulted in lower residual refractive error with improved visual quality metrics. This strategy is a viable option in patients with symptomatic cataracts and irregular corneas.

## 1. Introduction

Since its birth 25 years ago, laser assisted in situ keratomileusis (LASIK) has been using excimer laser ablation to reshape the cornea and correct refractive errors [1]. Those patients who underwent LASIK in early days are now presenting with age-related cataracts. Surgeries done in early years when the nomogram was being revised had resulted in few cases of irregular corneas. Small or decentered optical zones, irregular ablations, and central islands are associated with high corneal higher order aberrations (HOAs) [2, 3]. Obtaining optimal optical outcomes with cataract surgery in such cases is difficult. The dilemma of regularising the cornea first followed by cataract surgery or vice versa is also unresolved.

Topography-guided customised ablation treatment (T-CAT) outcomes improve visual acuity and quality in irregular corneas [4]. Refractive surgery complications, such as post-LASIK ectasia, decentered ablation, and small optical zones, have been successfully treated with this modality [5]. We hereby describe a method of customising the cataract surgeries in irregular corneas by doing a topoguided ablation to reduce corneal irregularity, assessing stability of keratometry and irregularity, followed by cataract surgery.

## 2. Methods

This was a prospective interventional case series adhering to the tenets of the Declaration of Helsinki with institutional ethical board clearance. Patients presenting with symptomatic cataracts and history of laser in situ keratomileusis (LASIK) in the same eye and giving informed consent were included. Exclusion criteria included preexisting ocular or chronic systemic disease, pregnant or nursing women, and one-eyed patients.

All patients underwent refraction, slit-lamp examination, indirect ophthalmoscopy, and topography using the Pentacam HR (Oculus Optikgeräte GmbH). Axial length measurements were by immersion biometry when optical biometry was not possible.

Eyes with post-LASIK corneal irregularities, with no evidence of ectasia on Pentacam HR, and with minimal corneal pachymetry of 400 microns underwent corneal regularisation using the topography-guided customised ablation treatment (T-CAT) software linked with the Allegretto Topolyzer system (WaveLight Laser Technologie AG, Germany). Adjunct corneal collagen cross-linking was not planned for this study subset of patients as the predicted minimum pachymetry was more than 380 microns in all cases. An average of eight maps with at least 90% of the data were taken by Allegretto Topolyzer system. The optical zone diameter was restricted to 5.5–6.0 mm after defining the target asphericity (*Q*-value) within a range of 0 to −0.6. Under topical anaesthesia (proparacaine 0.5%; Alcon Inc., Fort Worth, USA), the preexisting flap was raised and the planned laser ablation was performed, followed by balanced salt solution irrigation of the residual stromal bed. Postoperative regime was prednisolone acetate 1% eye drops (Pred Forte® prednisolone acetate ophthalmic suspension, Allergan, India) four times daily tapered weekly and moxifloxacin 0.5% eye drops (Vigamox®, Alcon Inc., Fort Worth, USA) four times daily for one week with lubricating eye drops (Optive®, Allergan, India) four times daily.

The IOL power was calculated after achieving keratometric stability defined as change of 0.2 dioptres or less in standard deviation of mean *K* over three consecutive visits. The IOL power chosen for implantation was the minimum IOL power obtained on American Society of Cataract and Refractive Surgery (ASCRS) post-LASIK calculator [6]. Patients underwent phacoemulsification by a single surgeon (M. K. Kummelil) using a temporal clear corneal incision.

### 2.1. Assessments

Holladay's equivalent keratometry reading (EKR) map on the Pentacam presents the mean equivalent keratometry readings and their standard deviations in zones around the corneal apex. We derived the zonal keratometric coefficient of variation (ZKCV) (dispersion of data points in a data series around the mean) from the zonal standard deviation and the zonal mean keratometry as follows:

 ZKCV = zonal keratometric standard deviation/zonal mean keratometry × 100

Efficacy assessments were the improvement in structural and functional parameters and safety was the percentage of eyes with loss of two or more lines of CDVA [7].

### 2.2. Statistical Analysis

SPSS version 17 was used for statistical analysis. The Shapiro-Wilk test checked normal distribution of continuous variables. Comparisons between groups were 2-sided to a significance of 0.05.

## 3. Results

This study included six eyes, three right and three left, of four patients, one male and three females, with a mean age of 41.25 ± 9.95 years. The mean duration after LASIK was 7.83 ± 2.40 years while the mean duration between T-CAT and cataract surgery was 1.5 months.

**Figure 1:**
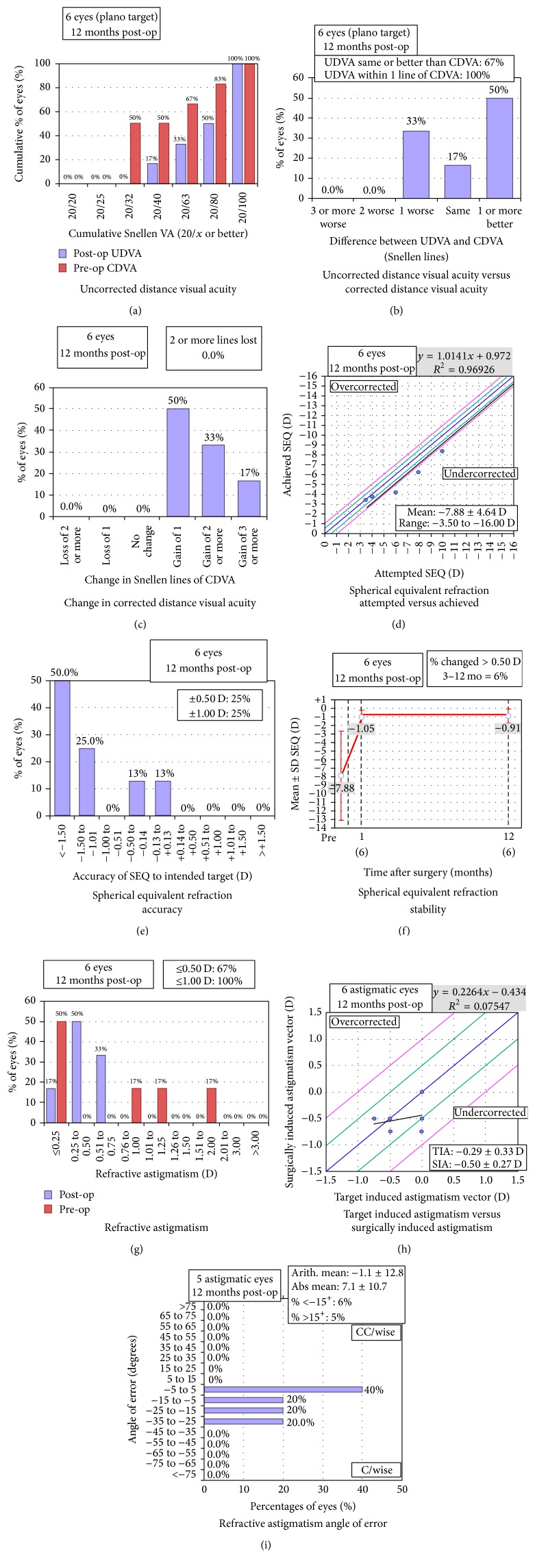
Standard refractive graphs showing (a) efficacy-uncorrected distance visual acuity (UDVA), (b) UDVA versus corrected distance visual acuity (CDVA), (c) safety-change in CDVA, (d) attempted versus achieved spherical equivalent refraction, (e) accuracy of spherical equivalent refraction, (f) amplitude of astigmatism, (g) stability of spherical equivalent refraction, (h) target induced astigmatism (TIA) versus surgically induced astigmatism (SIA), and (i) angle of error (C/wise stands for clockwise and CC/wise stands for counterclockwise).

The logmar uncorrected distance visual acuity (UDVA) at the time of presentation was 0.67 ± 0.25 (95%  CI 0.404 to 0.929) and following TPRK and cataract surgery showed a statistically significant improvement (Paired Samples Test *p* = 0.006) to 0.35 ± 0.14 (95% CI 0.205 to 0.495). The corrected distance visual acuity (CDVA) improvement from 0.38 ± 0.20 (95%  CI 0.169 to 0.598) to 0.18 ± 0.10 (95%  CI 0.080 to 0.287) was not statistically significant (Wilcoxon Signed Ranks Test *p* = 0.066). Standard refractive graphs have been shown in Figure 1.

The mean thinning in pachymetry after T-CAT was 33 ± 16 microns at pupillary center, 36 ± 17 microns at apex, and 32 ± 17 microns at the thinnest corneal thickness on Pentacam HR. Predicted Minimum pachymetry after treatment was 407 ± 16 microns with thinnest pachymetry being 380 microns. The RMS difference in the mean *K* showed a change of 2.12 D in the 1 mm zone and 1.40 D in the 4 mm zone. The mean reduction in the astigmatism (EKR 65% astigmatism) and the standard deviations of the *K* values were statistically significant from the 2 mm to 4 mm zone. The ZKCV showed a statistically significant reduction in the dispersion of measured keratometric data points around the mean keratometry for the 1 and 2 mm zones (Table 1). The changes in the Strehl ratio (ANOVA *p* = 0.043) were statistically significant while the change in mean HOAs (Friedman test *p* = 0.115) and area under the curve of the MTF curve (ANOVA *p* = 0.356) was not.

The mean IOL power used was 15.25 ± 3.52 D. In four of six (66%) eyes where the IOL power could be calculated before and after T-CAT, the root mean square (RMS) change in the ASCRS calculator minimum IOL power was 0.63 D and in the maximum IOL power was 1.30 D. The procedure was 100% safe as none of the eyes had loss of two or more lines of vision. Two of the six patients were within 0.5 dioptre sphere (DS) and four of the six patients were within 1 DS.

## 4. Discussion

Patients with myopic LASIK present with cataracts early as compared to general population [8]. Similar to the study done by Iijima et al., our patients presented with cataract at an average age of 42 years. Incidence of post-LASIK cataract is low, almost 1 in 100 cases of normal cataracts [8]. It is not common for patients who now present for cataract to have irregular astigmatism as a result of prior LASIK surgery and to the best of our knowledge no study has reported the management strategy in such cases.

Topographically irregular astigmatism has been classified as regularly irregular (asymmetric bow-tie or angled bow-tie or nonorthogonal astigmatism) and irregularly irregular (no recognizable pattern) [9]. Irregular astigmatism after LASIK depends on the amount of surgery the eye received, the time since surgery, the size and centration of the optical zone, and the occurrence of any intraoperative complications [10]. Irregularity is measured by assessing Zernike HOAs or Fourier irregularity. These tests do not give a measure of the variability of the keratometry. Therefore, a cataract surgeon has to estimate the impact of the preexisting corneal irregularity on the mean keratometry values needed for IOL power calculation. We describe a novel method of quantifying the corneal irregularity from the detailed Holladay Report by assessing the mean zonal keratometric coefficient of variation. As the mesopic pupillary diameter is 4 mm, we used values up to 4 mm zone for the analysis.

**Figure 2:**
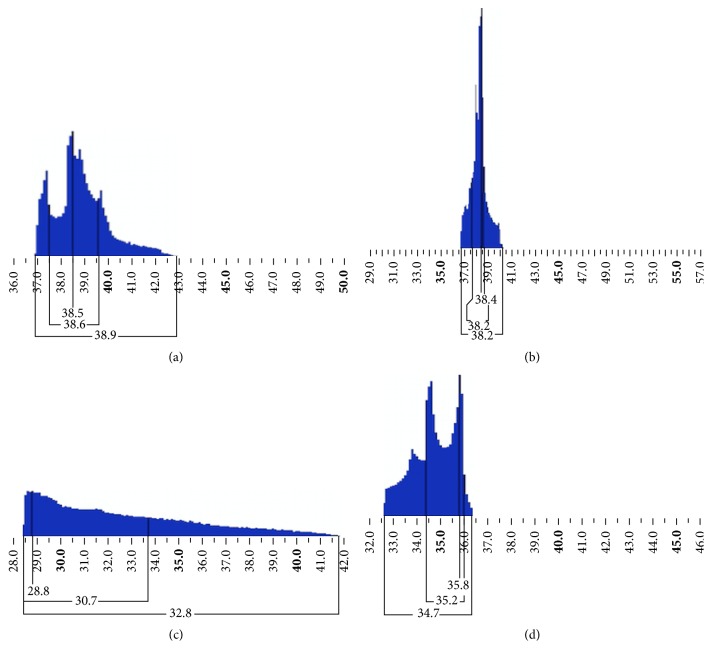
Equivalent *K* reading (EKR) histogram to demonstrate the improved outcome following T-CAT. (a) EKR histogram of case 3 is shown preoperatively with broader base extending from 37 D to 43 D with two peaks. (b) Postoperatively, the EKR histogram shows a tall peak with a narrow base extending from 37 D to less than 40 D. (c) The EKR histogram of case 6 is shown preoperatively with broader base extending from 28.5 D to 42 D. (d) Postoperatively, the EKR histogram shows a narrow base from 32.5 D to 36.5 D with two peaks.

Because of its unique therapeutic benefits in treatment of highly irregular corneas, T-CAT was our treatment of choice [11]. All our patients showed improved regularity of cornea after T-CAT as there was 45% in astigmatism, 52% reduction in standard deviation of *K* values, and 51% reduction in ZKCV till the 4 mm zone and this improvement was reflected in the improved EKR histogram (Figure 2).

**Figure 3:**
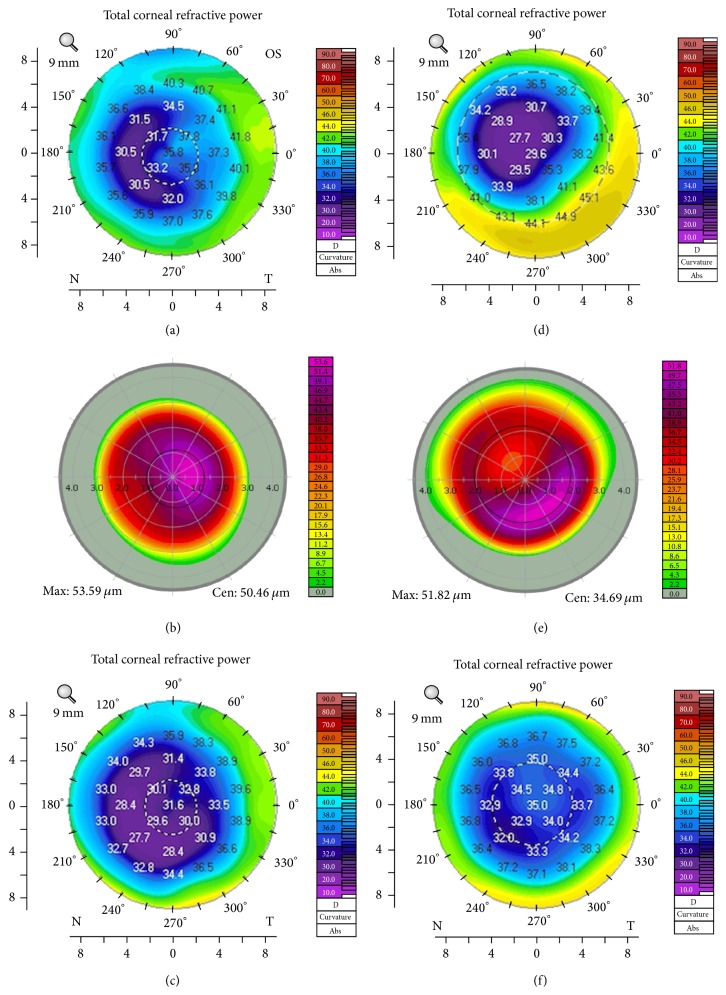
Two eyes with irregular corneas after LASIK. (a) The patient's cornea shows incomplete ablation with steep areas in the pupillary zone. (b) Intraoperative ablation profile used for treatment during topography-guided customised ablation treatment (T-CAT). (c) Postoperatively, there is flattening at the center of the cornea in the pupillary zone. (d) The patient's cornea with decentered ablation showing both flat and steep areas in the pupillary zone. (e) Intraoperative ablation profile used for treatment during T-CAT. (f) Postoperatively, there is steepening at the center of the cornea in the pupillary zone.

Following stabilization of keratometry, cataract surgery was performed with IOL implant power selected using the online ASCRS calculator targeting emmetropia [6]. In eyes with a central island, T-CAT would result in a relatively steeper central cornea resulting in a myopic shift in IOL power requirement and in eyes with decentered ablations T-CAT would result in relative central flattening cornea resulting in a hyperopic shift in IOL power requirement (Figure 3).

Our study is limited by its small sample size and nonrandomised comparison group. We also limited the duration after T-CAT to an average of 6 weeks because of the need for early visual rehabilitation. Possibility of corneal remodelling for longer duration persists. In absence of standard guidelines, we waited for 3 consecutive follow-ups showing stability in terms of mean *K* fluctuation.

Conventionally, a secondary LASIK or surface ablation has been the procedure of choice for postcataract refractive surprises [12]. However, the alteration in the mean keratometry values by the T-CAT would induce an unpredictable myopic or hyperopic shift in refraction depending on the nature of preexisting irregularity and treatment plan. Our strategy of treating the cornea by T-CAT first followed by cataract surgery is a safe and effective way of optimising results in these cases. It is a relatively rare clinical situation and our case series documents the outcome and benefits of a novel sequence of performing the procedure.

Numbers are bound to increase with time as more of the baby boomers/early LASIK patients and those with complications will require cataract surgery. So we are putting up the results for peer review and will expand the study to a case control/randomised control trial (RCT). If this strategy is seen to work in a RCT/case control trial then it can be considered as an option for the management of irregular astigmatism of all etiologies with coexisting cataract.

## Figures and Tables

**Table 1:** Mean ± standard deviation (95% confidence interval for mean) of the baseline variables, post-topoguided customised ablation treatment (T-CAT), change from baseline, and *p* values (paired sample tests).

	Baseline mean ± std. deviation (95% confidence interval for mean)	Post-T-CAT mean ± std. deviation (95% confidence interval for mean)	Change from baseline	*p* value (paired samples test)
1 mm mean *K*	34.56 ± 4.08 (30.28–38.84)	34.97 ± 2.94 (31.88–38.06)	2.12 ± 2.50	0.844
2 mm mean *K*	34.33 ± 3.62 (30.53–38.13)	34.74 ± 2.85 (31.75–37.72)	1.84 ± 2.27	0.834
3 mm mean *K*	34.61 ± 3.23 (31.23–38.00)	34.57 ± 2.73 (31.71–37.43)	1.72 + 1.65	0.980
4 mm mean *K*	34.97 ± 2.67 (32.16–37.77)	34.55 ± 2.55 (31.87–37.23)	1.40 ± 1.21	0.791
1 mm Astig EKR65	0.93 ± 0.53 (0.37–1.49)	0.47 ± 0.44 (0.01–0.93)	0.55 ± 0.51	0.133
2 mm Astig EKR65	1.40 ± 0.37 (1.01–1.79)	0.62 ± 0.42 (0.30–1.07)	0.82 ± 0.37	0.007
3 mm Astig EKR65	1.41 ± 0.43 (0.95–1.86)	0.59 ± 0.30 (0.27–0.90)	0.82 ± 0.45	0.003
4 mm Astig EKR65	1.48 ± 0.41 (1.05–1.91)	0.67 ± 0.30 (0.35–0.98)	0.81 ± 0.47	0.003
1 mm SD	0.84 ± 0.44 (0.38–1.30)	0.42 ± 0.14 (0.27–0.57)	0.46 ± 0.44	0.051
2 mm SD	1.28 ± 0.54 (0.71–1.84)	0.71 ± 0.24 (0.45–0.96)	0.58 ± 0.46	0.040
3 mm SD	1.66 ± 0.76 (0.86–2.46)	0.87 ± 0.29 (0.57–1.17)	0.79 ± 0.62	0.038
4 mm SD	2.03 ± 0.97 (1.01–3.0)	0.99 ± 0.38 (0.59–1.39)	1.04 ± 0.77	0.035
1 mm CV	2.45 ± 1.24 (1.14–3.75)	1.21 ± 0.40 (0.79–1.63)	1.36 ± 1.27	0.042
2 mm CV	3.84 ± 1.76 (2.00–5.69)	2.06 ± 0.79 (1.23–2.88)	1.81 ± 1.57	0.047
3 mm CV	4.98 ± 2.58 (2.28–7.68)	2.56 ± 1.03 (1.48–3.64)	2.42 ± 2.16	0.058
4 mm CV	5.99 ± 3.19 (2.64–9.33)	2.94 ± 1.34 (1.53–4.35)	3.05 ± 2.56	0.056

*K*: keratometry, EKR65: equivalent keratometry reading in 65% area, SD: standard deviation, and CV: coefficient of variance.
